# Early Growth Response 3 (Egr3) Is Highly Over-Expressed in Non-Relapsing Prostate Cancer but Not in Relapsing Prostate Cancer

**DOI:** 10.1371/journal.pone.0054096

**Published:** 2013-01-14

**Authors:** Rebecca Pio, Zhenyu Jia, Veronique T. Baron, Dan Mercola, UCI NCI SPECS consortium of the Strategic Partners for the Evaluation of Cancer Signatures, Prostate Cancer

**Affiliations:** 1 Department of Pathology and Laboratory Medicine, University of California Irvine, Irvine, California, United States of America; 2 Vaccine Research Institute of San Diego, San Diego Science Center, San Diego, California, United States of America; Florida International University, United States of America

## Abstract

Members of the early growth response (EGR) family of transcription factors play diverse functions in response to many cellular stimuli, including growth, stress, and inflammation. Egr3 has gone relatively unstudied, but here through use of the SPECS (Strategic Partners for the Evaluation of Predictive Signatures of Prostate Cancer) Affymetrix whole genome gene expression database we report that Egr3 mRNA is significantly over-expressed in prostate cancer compared to normal prostate tissue (5-fold). The Human Protein Atlas (http://www.proteinatlas.org), a database of tissue microarrays labeled with antibodies against over 11,000 human proteins, was utilized to quantify Egr3 protein expression in normal prostate and prostate cancer patients. In agreement with the SPECS data, we found that Egr3 protein is significantly increased in prostate cancer. The SPECS database has the benefit of extensive clinical follow up for the prostate cancer patients. Analysis of Egr3 mRNA expression in relation to the relapse status reveals that Egr3 mRNA expression is increased in tumor cells of non-relapsed samples (n = 63) compared to normal prostate cells, but is significantly lower in relapsed samples (n = 38) compared to non-relapse. The observations were confirmed using an independent data set. A list of genes correlating with this unique expression pattern was determined. These Egr3-correlated genes were enriched with Egr binding sites in their promoters. The gene list contains inflammatory genes such as IL-6, IL-8, IL1β and COX-2, which have extensive connections to prostate cancer.

## Introduction

The early growth response (EGR) transcription factors have long been implicated in multiple cellular processes important to cancer, including apoptosis, differentiation, proliferation, growth inhibition, and inflammation [1]–[5]. EGR transcription factors are induced rapidly and transiently in response to diverse stimuli such as growth factors, cytokines, phorbol esters (TPA) and ionizing radiation, and regulate a diverse array of genes in response to these stimuli [1], [6]–[8]. The EGR family is comprised of Egr1, Egr2, Egr3, and Egr4 [9] and all family members bind to the same EGR response DNA element (ERE), GCGG/TGGGCG, through three conserved zinc finger DNA binding domains [10].

Egr1 is the best studied member of the transcription factor family. Numerous studies have detailed its tumor suppressor functions and consequently its down-regulation in breast, lung, and glial cancers [11]–[13]. Interestingly, Egr1 has been shown to act as an oncogene in prostate cancer. Multiple investigators have reported the over-expression of Egr1 mRNA and protein in prostate cancer [14]–[15]. The TRAMP and CR2-T-Ag mouse models of prostate cancer were utilized to further examine the functional role of Egr1 in the initiation and progression of the disease. Egr1-null mice that were crossbred with either cancer model showed delayed progression from prostatic intraepithelial neoplasia (PIN) to invasive carcinoma [16].

Despite the well characterized functions of Egr1, far less is known about the other transcription factors in the EGR family such as Egr3. Several studies detail the function of Egr3 in neural development, specifically muscle spindle development, sympathetic neuron differentiation, and response to environmental stress (sound, handling, and novel situations) [17]–[20]. Egr3-deficient mice exhibit sympathetic dysautonomia and severe sensory ataxia [17], [20], whereas Egr1-deficient mice exhibit no apparent behavioral or developmental problem [21]. Egr3 knockout mice have not yet been utilized to study the role of the transcription factor in cancer, however recent reports have used cell culture models and gene expression data to study Egr3 function in several areas important to cancer. Thus, Egr3 is up-regulated by vascular endothelial growth factor (VEGF) in human umbilical vein endothelial cells (HUVECS) [22], [23], and knockdown of Egr3 in these cells results in a reduction of VEGF-induced proliferation, migration, and tubulogenesis [23]. In addition to its role in angiogenesis, several reports have investigated the role of Egr3 in breast cancer, where it was found to be an estrogen-responsive gene whose immunoreactivity is positively associated with estrogen receptor α (ERα) status, lymph node status, and distant metastasis [24], [25].

Much work remains to be done, however, on unraveling the role of Egr3 in other types of cancer. Based on our knowledge of Egr1 and the common DNA binding characteristics of all EGR transcription factors, we hypothesize that Egr3 does indeed play a role in either the formation or progression of prostate cancer. Here we report the over-expression of Egr3 mRNA and protein in prostate cancer compared to normal prostate tissue. In addition to studying the over-expression of Egr3, we also used the extensive prostate gene expression datasets from the University of California-Irvine SPECS program to examine the expression pattern of Egr3 in prostate cancer patients with known relapse status, and found that Egr3 expression in tumor cells is lower in tumors that relapse compared to tumors that do not relapse. This expression pattern: low Egr3 mRNA in normal prostate versus high expression in cancer, and distinction between relapse and non-relapse, was confirmed with the independent prostate cancer gene expression dataset from the University of Pittsburg [26]–[28].

Using the Human Protein Atlas, we also detail a unique in-silico method for investigating the over-expression of Egr3 protein in prostate cancer. The combined results indicate that Egr3 is a biomarker of poor outcome prostate cancer. Moreover, a cohort of highly correlated genes exhibits a similar expression pattern, and the members of this cohort are candidate Egr3-regulated genes.

## Materials and Methods

### Prostate Tissue Samples

Prostate samples were acquired by informed consent according to University of California, Irvine Institutional Review Board (IRB)-approved and HIPAA-compliant protocols. Tissue acquisition was part of the NCI-SPECS program at the University of California, Irvine. Prostate cancer tissues were collected at the time of prostatectomy, reviewed by a pathologist, and snap-frozen in liquid nitrogen. Tissue tracking sheets were maintained to record the elapsed time from surgery to freezing (average 2.8 hours). Normal prostate samples were either snap-frozen in liquid nitrogen from fresh biopsy cores or snap-frozen after collection from the rapid autopsy program at Sun Health Research Institute (Sun City, AZ), a SPECS consortium member. The data consists of 19 normal prostate samples from 13 individuals (12 from normal biopsy and 7 from rapid autopsy) and 108 prostate cancer samples from 84 individuals (**Table 1**). Outcome parameters and relevant clinical data including an extensive history were accumulated over an 11 year period and maintained in the SPECS relational data base with a data dictionary of over 250 items. The outcome parameter “relapse” refers to biochemical relapse, the rise in PSA levels over 0.2 ng/ml following a prior post-op PSA that was below threshold for the test. Non-relapse means that no biochemical relapse was observed in the patient during the clinical follow-up time frame. Note that all tumor samples were collected at the time of prostatectomy, so relapse status was determined after the samples were already collected. Relevant clinical and demographic values are summarized in **Table 1**.

**Table 1:** Demographical information of 97 patients used for the analysis of Egr3 in relapse and non-relapse prostate cancer.

Normal Prostate Samples	
Age	# of patients
40–55	4
56–65	2
66–75	2
76+	5
**Prostate Tumor Patients**	
**Age**	**# of patients**
40–55	17
56–65	46
66–75	21
**Race**	**# of patients**
Caucasian	36
African American	8
Hispanic	4
Other : (filipino, native american, korean)	3
Unknown	33
**Pre-Operative PSA**	**# of patients**
<5	25
5.1–7	18
7.1–9	11
>9	19
Unknown	11
**Gleason Sum**	**# of patients**
5–6	19
7–8	34
9–10	6
NA	25
**Stage**	**# of patients**
2	8
3	3
2a	3
2b	14
2c	18
3a	6
3b	2
unknown/NA	30

* 84 prostate cancer patients provided 108 arrays and 13 normal prostate donors provided 19 arrays.

### Affymetrix Gene Expression Arrays and Statistical Analysis

RNA from the prostate tissue samples was analyzed using Affymetrix gene expression arrays. The RNA was prepared from the fresh frozen tissue by sectioning tissue blocks with a cryostat and purifying total RNA directly from the accumulated frozen sections. Purified RNA was hybridized to either Affymetrix U133 Plus2.0 or U133A gene expression arrays, and the arrays were processed according to Affymetrix protocol. The intensity data used here is available from public sources (GEO GSE17951 and Geo GSE8218, respectively).

Normalized Affymetrix intensity values were used to compare Egr3 (Affymetrix probe ID 206115_at) expression in whole samples of normal prostate and prostate cancer tissue. Normalized values for the two sample types were compared using a student’s t-test. LIMMA (Linear Models for Microarray Data) analysis from Bioconductor was implemented in the R environment to detect differentially expressed genes [29].

In order to analyze the cell type specificity of expression of Egr3 and correlating genes, we used a multiple linear regression (MLR) model to describe the relationship between the observed Affymetrix gene expression level 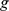 and cell type composition for four cell types in relapsed and non-relapsed cases:
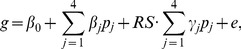
(1)where 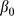 is the intercept, 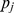 is the percentage of cell type j as determined by a panel of pathologists, 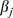 is the coefficient for contribution from cell type 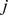, 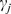 is the deviation or change of 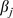 for cell type 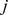 when the disease relapses. 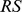 is the indicator variable for relapse status. 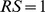 if subject undergoes relapse and 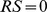, otherwise. 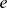 is the random error with assumed distribution 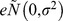. This MLR model was used to fit the data to estimate the expression coefficients, 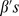 and 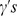, where 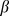 is the cell type-specific expression coefficient and γ is the difference in cell type-specific expression between relapse and non-relapse cases. Thus, *β*_j_ measures the expression of a gene by cell type j of non-relapsed cases and *γ*_j_ is the change in *β*_j_ in relapsed cases. For MLR, β_0_ is the mean of expression of all genes for all cell types and outcome status (RS). Therefore β_j_ coefficients <0 indicate that expression of gene j in a given cell type is less than the mean β_0_, while β_j_ coefficients >0 indicate increased expression compared to β_0_. Similarly, γ_j_ <0 indicates that expression of gene j is reduced in relapsed prostate cancer compared to non-relapsed prostate cancer whereas γ_j_ >0 indicates that expression of gene j is increased in relapsed prostate cancer compared to non-relapsed disease. The significance of γ_j_ was determined by t-test, based on the error σ_j_ of γ_j_. The accuracy of cell type-specific expression contribution coefficients, β, has been validated [30].

### Human Protein Atlas

Immunohistochemistry images were downloaded from the publicly available Human Protein Atlas (HPA) (http://www.proteinatlas.org). HPA version 8.0 is a database of tissue microarray (TMA) images labeled with antibodies against 11,250 human proteins [31]. The tissue microarrays consist of sections from 46 normal human tissues and 20 different types of human cancer. There are a maximum of 24 prostate cancer images (from 12 patients) and 3 normal prostate images (from 3 donors) per antibody, although image number may vary depending on the antibody analyzed. The HPA images analyzed were prostate sections labeled with either Egr3 (HPA006206), PSMA (CAB001451, Leica Microsystems, Wetzlar, Germany), or PRLH (HPA014768) antibodies.

The HPA images were analyzed and labeling intensity was quantified with the Aperio ImageScope software (Vista, CA) (**Figures S1 and S2**). The Positive Pixel Count Algorithm version 9.1 was used to quantify the amount of antibody binding and to obtain pseudocolored TMA images. In order to quantify labeling of particular structures such as stroma or tumor acini, the intensity threshold values set by the Aperio software were used, according to the manufacturer suggestions.

Pixel intensity is defined as a measure of the brightness of the pixel, or the amount of light transmitted through the slide. The lower the intensity value is, the darker the staining is owing to increased absorbance A = −log I_obs_/I_ref_, where I_obs_ indicates transmitted light intensity and I_ref_ is the incident light intensity. Intensity thresholds were set at 220, 175, 100, and −1 for the weak, medium, strong, and “negative” pixels, respectively. Pseudocolors of blue, yellow, orange, and red were applied to the negative, weak, medium, and strong pixels, respectively.

Normal prostate and prostate cancer tissue were analyzed with the positive pixel count algorithm. To compare the density of staining in normal and prostate cancer tissues, the NSR (number of strong positive pixels ratio) was used. The number of strongly labeled positive pixels is divided by the total number of positive pixels to normalize each sample to the area under consideration, thereby providing labeling intensity of a selected cell type per unit area of tissue.

The positive pixel count algorithm was also used to compare protein expression in the stromal and epithelial components of the prostate tissue. A mouse-guided pen tool was used to outline 10 stromal and 10 gland areas per TMA section. The 10 sampling areas were chosen randomly whenever possible, within the constraints of tissue size and composition.

Constraints in tissue size: the total tissue area was only 1 mm in diameter, and the edges of each tissue section were avoided because staining in these areas tend to be affected by the processing of the TMAs. In addition, we used sampling areas that did not touch each other.

Constraints in tissue composition: some tissue sections contained many glands, and when this was the case the sampling areas were chosen randomly. Other sections contained much fewer gland areas, reducing our choice to the glands that were present. The stromal sections, on the other hand, were all chosen at random since the tissue sections had numerous stromal areas.

A snapshot was taken of each sample chosen at random and used to verify that the samples used for analysis were representative of the whole section.

The results were averaged and used for determination of significant differences compared to normal prostate tissue.

### Egr3-correlated Genes

Pearson’s Correlation Coefficient (R) and the associated probability and slope were calculated in the R environment for the correlation of expression of each gene on the Affymetrix array platform with the expression of Egr3. The genes with *p* values ≤0.001 based on Pearson’s correlation analysis and a Pearson correlation coefficient ≥0.45 were selected as defining Egr3-correlated genes. Egr3-correlated genes for the main SPECS Affymetrix U133 Plus2.0 dataset (127 prostate samples) used in this study, and Egr3-correlated genes for an additional SPECS gene expression dataset (136 prostate samples) based on the Affymetrix U133A platform, were calculated. The overlapping genes between datasets that met the significance cutoff values were taken as the final list of Egr3-correlated genes. The slope of the regression line was also used to characterize the adherence of each correlation to Egr3 expression and is reported in Table S1.

### Statistical Simulation

Several simulations were carried out in order to assess random occurrence. In general the frequency of occurrence of candidate probe sets in a class was compared to the frequency of occurrence by random selection of an equal number of probe sets from the rest of the array, i.e. from all probe sets excluding the candidate probe sets using the R program. Random selection was repeated 10,000 times and the average random frequency of occurrence compared to the observed frequency of the candidate probe sets was used to establish the probability of random occurrence.

### Transcription Factor Binding Site and Gene Network Analysis

Genomatix (Munich, Germany) MatInspector software was used to determine transcription factor binding sites for promoter regions 1500 bases upstream to 1000 bases downstream of the transcription start site (tss). The transcription factor binding sites (weight matrices) library and vertebrate general core promoter elements (0.75/Optimized) matrix group were used to calculate binding sites for each promoter. Based on the MatInspector background model for occurrences of V$EGRF (Transfac annotation for the vertebrate Egr Family members Egr1, Egr2, Egr3, CKROX, and WT) binding sites, a 10,000×simulation was performed in the R environment to compare the background percentage (62.7%) of V$EGRF binding sites as determined by MatInspector to the observed percentage (99%). *p*-value = the number of times the expected was greater than the observed/10,000 using Genomatix. The simulation *p*-value provides an estimate of false discovery.

MetaCore (Thomson Reuters, New York, NY) pathway analysis software was used to analyze connections between Egr3-correlated genes. Transcription factor enrichment analysis and protein function enrichment analysis were calculated by MetaCore using a false discovery rate (FDR) of 0.05 and a background model of reported protein-protein or protein-DNA interactions. All *p*-values reported for the MetaCore analysis come directly from MetaCore’s calculations based on a hypergeometric distribution. The web-based gene ontology program DAVID [32], [33] (http://david.abcc.ncifcrf.gov/home.jsp) was also used to analyze functional connections between Egr3-correlated genes.

### External Validation Dataset

The publicly available GEO dataset GDS2545 was used as an external validation of Egr3 and Egr3-correlated genes identified in the SPECS dataset. GDS2545 consists of 18 samples of normal prostate from donors, 63 samples of normal prostate adjacent to tumor, 65 samples of primary prostate cancer, and 25 samples of metastatic prostate cancer. All samples were hybridized on Affymetrix U95A arrays. Chandran et al. [27] and Yu et al. [26] report Affymetrix Egr3 expression values in supplementary information and in tables of gene expression values. Egr3-correlated gene analysis was carried out using this dataset. The top 150 Egr3-correlated probe sets as judged by Pearson’s correlation *p*-value consists of 121 unique genes, which were compared to the 80 unique Egr3-correlated genes identified in the SPECS dataset yielding an overlap of 43 unique genes. A 10,000×simulation was performed in the R environment to determine the likelihood of this overlap occurring by chance (*p* = 0.0001).

### Cell Culture

M12 human prostate cancer cells were maintained in serum-free RPMI medium supplemented with L-glutamine, 10 ng/ml epidermal growth factor, 0.1 µM dexamethazone, 5 µg/ml insulin, 5 µg/ml transferrin, 5 ng/ml selenium, 0.05 mg/ml gentamicin and 2.5 µg/ml amphotericin B, as described in [34].

### Stable Knockdown of Egr3 Expression in M12 Cells

M12 cells were plated the day before transfection at a density of 750,000 cells/well in 6-well plates, in antibiotic-free growth medium containing 2% Fetal Bovine Serum. Cells were transfected with 3 µg shRNA scramble plasmid (shSCR) or shEgr3 plasmid (SA Biosciences, Valencia CA) with 6 µl Lipofectamine 2000 (Invitrogen, Carlsbad CA) according to the manufacturer's instructions. Puromycin (1 µg/ml) was added three days later for selection of transfected cells. Single cell clones were isolated using standard methods. Stably transfected cells (shSCR-M12 and shEgr3-M12) were then maintained in M12 growth medium supplemented with puromycin.

### Western Blot Analysis

Cells were washed twice in phosphate buffered saline and lysed in RIPA buffer in the presence of protease inhibitors. The lysate was sonicated briefly and cleared by centrifugation at 13,200 rpm at 4°C. Protein concentration was determined using BioRad Protein Assay. Proteins were subjected to SDS-PAGE electrophoresis followed by transfer to PVDF membrane. Membranes were blocked in 5% milk (w/v) in Tris-buffered saline containing Tween-20 0.5% (TBST) for 2 hours. Egr3 antibody (sc-191 Santa-Cruz Technology, Santa Cruz CA) was incubated overnight at 4°C, followed by 3 washes in TBST. The membranes were incubated with HRP-conjugated antibodies for 1 hour at room temperature (RT). After 3 washes in TBST, membranes were incubated with HyGlo-HRP Chemiluminescent kit (Denville Scientific, Metuchen NJ). Membranes were stripped and reprobed with anti-actin antibodies (Santa-Cruz).

### Quantitative RT-PCR

Total RNA was extracted from shSCR-M12 (scramble control) or shEgr3-M12 cells using the RNeasy Plus kit (Qiagen, Valencia CA) as per instructions, and resuspended in water. RNA integrity was assessed by electrophoresis on formaldehyde agarose gel. One µg RNA was converted to cDNA using the Quanta qScript cDNA Super-mix (5 min at 25°C; 30 min at 42°C; 5 min at 85°C) in a thermocycler. Real-time qPCR was performed on the ABI Prism 7900HT (Applied Biosystems, Life Technologies, CA) using standard parameters. Each sample was run in four replicates with dissociation curve analysis. Differences in mRNA levels were analyzed using the 2^−ΔΔCT^ method. GAPDH was used to normalize samples. Primer sequences are provided in **Figure S4.**

### Reporter Assay

The human IL8 promoter (bases −262 to −55) cloned into the pGL3 luciferase plasmid and the control pRL-SV40 Renilla luciferase plasmid were a kind gift from Dr. Carlotta Glackin (City of Hope, Los Angeles, CA) and have been described in Li et al. [35]. The IL6-pGL3 Luciferase plasmid containing the IL6 promoter was a gift from Dr. Steve Cole (UCLA, CA) and has been described in Eickelberg et al. [36]. The shSCR-M12 cells and shEgr3-M12 cells were plated at a density of 20,000 cells/well in a white 96-well plate the day before transfection, and grown in antibiotic-free, phenol red-free growth medium. Cells were transfected with IL6-pGL3 or IL8-pGL3 plasmids and the renilla luciferase plasmid (to normalize for efficacy of transfection and cell numbers). FuGene transfection reagent (Promega, Madison, WI) was used as per the manufacturer’s instructions with 160 ng pGL3 plasmid, 40 ng pRL plasmid and 0.6 µl FuGene. Two days later, Dual-Glo Luciferase Reagent (Promega) was added to the growth medium (1∶1 vol/vol) and incubated for 10 min RT. Plates were read using a Dynatech ML-3000 luminometer. The reaction was quenched by addition of the Dual-Glo Stop & Glo reagent, which also initiates the renilla luciferase reaction, for 10 minutes at RT before reading. The ratio of Firefly to Renilla luminescence was calculated for each well.

## Results

### Cell Type-specific RNA Expression of Egr3 is Increased in Prostate Cancer

**Figure 1:**
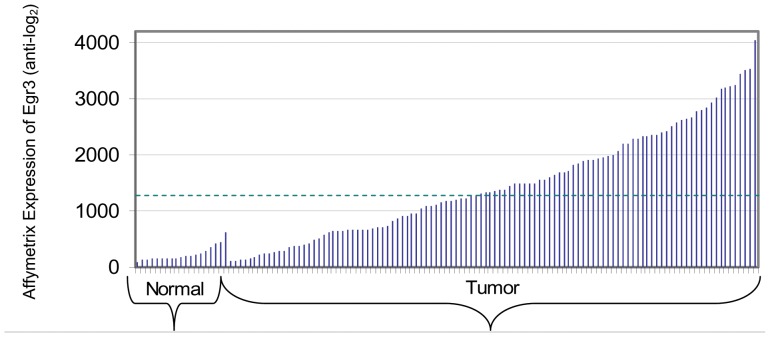
Normalized Affymetrix expression of Egr3 (probe set 206115_at) in the SPECS U133Plus2.0 dataset consisting of 19 normal prostate samples and 108 prostate cancer samples. Affymetrix expression is plotted on a linear scale where the anti-log_2_ of each patient’s Egr3 expression value is plotted on the y-axis. The dashed line at 1292 denotes the mean Egr3 intensity value for all samples.

We analyzed Affymetrix whole genome expression data from normal and cancerous prostate tissue to determine if Egr3 is differentially expressed. The Affymetrix U133 Plus2.0 gene expression data consists of 19 normal prostate samples (obtained from 13 donors) and 108 prostate tumor samples (obtained from 84 prostate cancer patients; several samples from the same patients were present in the gene expression data set). Patient demographical information for the 84 cancer patients and the 13 normal tissue donors is summarized in **Table 1**. Analysis of the normalized expression data from the 127 prostate samples revealed that Egr3 (probe set 206115_at) is significantly over-expressed in prostate cancer compared to normal prostate tissue. Mean expression in prostate cancer is 5.35 fold higher than in normal prostate tissue and a student’s t-test (*p* = 2×10^−15^) confirmed that this difference is highly significant. Analysis of median expression values also reveals a significant difference in Egr3 expression between normal prostate and prostate cancer samples (data not shown). A histogram of Egr3 expression across all patients is shown in **Figure** **1**. Egr3 expression values are displayed as Affymetrix intensity values rather than plotted on a log2 scale to better display their range. Since the average ages of the tumor-bearing cases differ from that of the normal cases (**Table 1**), the Affymetrix expression difference for Egr3 was compared to the list of previously determined age-related changes of Jia et al. [37] based on a comparison of expression data for 15 normal prostate biopsy samples with an average age of 54 years to those for the 13 rapid autopsy samples with an average age of 84 years. Of the 3400 probe sets yielding significant differences identified in this comparison, probe sets for Egr3 were not included. These results indicate that the increased expression of Egr3 is not associated with the age difference between normal donors and prostate cancer patients and is associated with one or more properties specific to tumor-bearing prostate tissue.

### Validation at the Protein Level with an External Data Set

Although Egr3 is up-regulated at the mRNA level, it may be the Egr3 protein level that is important to the function of Egr3 in prostate cancer. To complement the gene expression data we utilized an *in silico* approach to analyze Egr3 protein levels in prostate tissue. The Human Protein Atlas (HPA) is a collection of tissue microarray data for 20 different types of cancer and 46 normal tissues (http://www.proteinatlas.org). Tissue morphology and protein expression patterns for the HPA patient samples were verified by a board-certified pathologist. HPA provides an indication of protein expression in cancer compared to normal tissue counterparts. The HPA images were downloaded for further quantitative analysis. The available prostate tissue immunohistochemistry images for Egr3 staining consist of 20 prostate cancer samples from 11 patients and 3 normal prostate samples from 3 donors.

**Figure 2:**
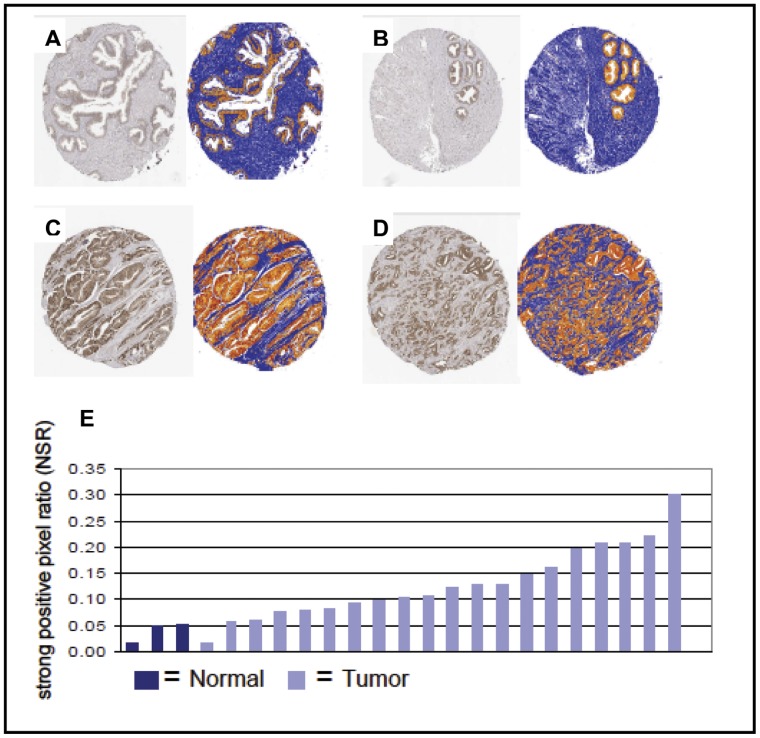
Human Protein Atlas immunohistochemistry using anti-Egr3 antibodies (left) and Aperio ImageScope pseudocolored prostate sections (right) based on thresholding as described in Materials and Methods. **A–B**: HPA normal prostate, patients 2098 and 2472, respectively. **C–D**: HPA prostate cancer samples, patients 3303 and 3744, respectively. All additional available HPA cases are shown in the supplement information. E: histogram of strong positive pixel ratio (NSR) for normal and prostate cancer patients.

We used the Aperio ImageScope positive pixel count algorithm to quantify the intensity of Egr3 staining (HPA006206). Pseudocolors were applied as described in Methods and are shown in **Figure** **2**. Although only four threshold levels were used, the pseudocolored images clearly illustrate that Egr3 staining intensity separated the epithelial-containing glandular structures from other features such as the stroma (**Figure** **2** **)**, indicating that the simple thresholding method adequately separated glandular elements from all else. The stroma was mostly devoid of Egr3 staining. For both normal and tumor samples, the predominant anti-Egr3 staining pattern was uniformly epithelial. Within epithelial cells, Egr3 staining was observed in the cytosol- membrane compartments and weakly in the nucleus.

Although transcription factors may be expected to localize mostly to the nucleus, this staining pattern is not unique to Egr3 in the Human Protein Atlas. As an example, we looked at the staining of a well studied transcription factor, c-fos. Staining for c-fos (HPA018531) is mostly nuclear, but also appears at a cytoplasmic-membrane localization in four out of ten prostate cancer tissues that stained positive for c-fos.

It is also possible that Egr3 localization is altered in pathological states, as is the case for transcription factor and tumor suppressor p53, for example. Indeed, wild-type p53 accumulates in the cytoplasm of tumor cells in inflammatory breast cancer or neuroblastoma, leading to its functional inactivation [38]–[40]. It could be interesting, in the future, to confirm the cytoplasmic/membrane localization of Egr3 and its relevance.

To quantify Egr3 staining we selected an average of 10 gland and 10 stroma regions in each patient sample. Each of the 10 regions was delineated using a mouse-guided pointer so that only regions of pure histological feature such as glands and gland-like tumor formations were included. The software recognized gland lumen and pseudolumen faithfully and excluded pixels of these regions so long as care was taken to exclude lumen with debris. The resultant Egr3 staining intensity values from all 10 areas for tumor and stroma were normalized separately. For this step the number of weak, medium and strongly stained pixels were weighted (weak = 1, medium = 2, strong = 3). The weighted values were summed and divided by the total number of pixels (negative and positive) to normalize each sample to the total area analyzed, yielding labeling intensity per unit area of gland, tumor, or stroma. Comparison of the normalized values by t-test showed that Egr3 protein expression is strongly epithelial and weakly stromal. The average value for stroma is 0.13 weighted positive pixels/total pixels while the average value for normal glands is 1.57 weighted positive pixels/total pixels (*p* = 1.65×10^−20^).

The Aperio ImageScope positive pixel count algorithm was also used to compare strong Egr3 staining in the normal and prostate cancer samples. A distribution of the NSR (number of strong positive pixels ratio) is shown in **Figure 2E**. The average NSR for normal prostate was 0.039 strong positive pixels/total positive pixels, while the average NSR for the prostate cancer samples was 0.13 strong positive pixels/total positive pixels, (*p = *0.00047, t-test). Both normal and cancer samples exhibited Egr3 staining of the prostate glands; however the cancer samples had a significantly higher number of pixels strongly labeled with Egr3 antibody. As a positive control for the ImageScope analysis PSMA (prostate specific membrane antigen) labeling of prostate tissue was quantified, and as a negative control PRLH (prolactin releasing hormone) labeling of prostate tissue was quantified. For PSMA, the immunohistochemistry HPA contained 13 samples from 7 patients. We selected one tissue section from each patient for a total of 7 PSMA immunohistochemistry images. As expected the prostate tissue samples exhibited significantly increased PSMA staining in the prostate cancer samples compared to normal prostate (*p* = 0.001) and antibody staining was predominantly epithelial. We haphazardly selected 4 PRLH-stained tissue sections from the 22 available prostate cancer tissue sections. PRLH staining was negligible in the 4 selected prostate cancer samples and the 2 available normal prostate samples, consistent with its selection as a negative control.

The HPA immunohistochemistry images were also reviewed by a pathologist and assigned Gleason scores. No significant association was found between Egr3 staining intensity and Gleason grade (Pearson Correlation Coefficient = 0.35; *p* = 0.29, two tailed); however the range of Gleason scores was minimal (6 through 8) with the majority of scores being 6 and 7.

Based on our analysis of the immunohistochemical labeling intensity of Egr3 in tissue sections, we conclude that Egr3 protein is more strongly expressed in prostate tumor epithelial tissue than in normal prostate glands, which is in good agreement with the RNA expression analysis.

### Egr3 RNA Expression in Prostate Cancer Cells is Specifically Correlated with Clinical Outcome

Because of the extensive clinical follow-up that has been recorded through the UCI-SPECS project, we were able to examine whether Egr3 expression is correlated with the outcome status of the patients. We asked whether the gene expression changes in patients who either did not relapse or did relapse after prostatectomy indicate whether Egr3 expression correlates with the severity of disease. Moreover, this analysis can be done on a cell type-specific basis [30].

Importantly, the percent cell composition (% tumor, stroma, BPH) for each sample in the gene expression dataset was determined by a panel of four pathologists. We formerly reported a multiple linear regression model that utilizes the percent cell composition to calculate how much each cell type component contributes to the overall expression value for each gene on the array [30]. By combining the known relapse status and the cell type-specific expression value for each gene the differential gene expression value between relapse and non-relapse cases was calculated for the three major cell-types that make up the tissue samples (eqn. 1, Materials and Methods). The differential gene expression corresponds to the gamma (γ_j_) coefficients of equation 1 for each cell type of the MLR model. γ_j_ is equal to the difference in cell type-specific expression in relapse patients minus the cell type-specific expression in non-relapse patients (γ_Egr3_ = β_nonrelapse_ - β_relapse_). We found that Egr3 has a γ_j_ value for tumor cells of -1.96 (*p = *0.023), indicating that Egr3 mRNA expression in tumor cells is negatively correlated with relapse status, and this is statistically significant (**Table** **2**). Egr1 expression is also lower in relapse compared to non-relapse (γ_j_ value -0.784; *p = *0.017), although the differential is not as high as that of Egr3. In contrast to tumor-specific expression, the γ_j_ coefficients for stroma or benign prostate epithelial cells were not significantly different (**Table** **2**). Indeed, of the 54,000 probe sets of the U133plus2 array, Egr3 exhibits the 40^th^ most negative γ_j_ tumor value observed. The γ_j_ tumor value for Egr3 is therefore both large magnitude (highly negative) and statistically significant, indicating that Egr3 is a candidate prognostic marker of poor outcome prostate cancer.

**Table 2:** Cell type-specific expression coefficients* for Egr transcription factors in relapse and non-relapse prostate cancer (n = 108 arrays).

		β_j_ relapse	β_j_ non-relapse	γ_j_	Probability γ_j_ *
Egr3	**Tumor**	−0.796	1.125	−1.921	**0.023**
	**Stroma**	2.638	1.783	0.856	0.272
	**BPH**	−0.601	−8.409	7.808	0.365
Egr1	**Tumor**	−1.088	−0.304	−0.784	**0.017**
Egr2	**Tumor**	0.072	0.994	−0.923	0.139

* Probabilities of β_j_ were all <0.05 and are not shown; equations for calculating β_j_ are defined in the Materials and Methods. βj are expressed relative to the mean expression of all probe sets of the array.

On the other hand, Egr3 was not significantly correlated with other clinical parameters such as Positive Surgical Margin, Age, Gleason score, Stage or Pre-operative PSA level.

### Promoters of Egr3-correlated Genes are Enriched in Egr Regulator Sequences

Because Egr3 is a transcription factor we sought to determine whether other transcripts were specifically elevated in correlation with the levels of Egr3 transcript in the prostate cancer tissues. Two gene expression datasets were used to assemble a list of genes whose expression patterns positively correlate with Egr3’s pattern, the Affymetrix U133 Plus2.0 dataset mentioned above and an Affymetrix U133A dataset also from the SPECS study (**Table S1)**. To narrow the correlating gene list, only genes with a correlation *p*-value ≤0.001 and a Pearson correlation coefficient (R_p_) ≥0.45 were chosen. These selection criteria were applied to both Affymetrix datasets, and the overlap of correlating genes between the U133 Plus2.0 dataset and the U133A dataset was determined yielding 98 probe sets. The correlating genes from the smaller list U133A overlapped with the U133Plus2.0 genes by 75%. Of the 98 Affymetrix probe sets common between the two datasets, there are 80 unique annotated genes whose mRNA expression correlates with Egr3 expression across the 263 prostate samples (136 samples from U133A and 127 samples from U133Plus2.0). In addition to using the Pearson’s correlation coefficient as a measure of the association between Egr3 and each correlating gene, we also used the slope of this line as a measure of how tightly the expression of the correlating gene follows Egr3 expression. **Table S1** is sorted by the regression line slope. 37 genes exhibited a slope >0.5 indicating a close relationship with the expression of Egr3.

Because of the general nature of correlating genes we expect that their expression patterns will follow that of Egr3. Indeed, 90 out of 98 probe sets display higher expression in non-relapse tumor samples than they do in relapse tumor samples. Of the correlating genes, 14 probe sets (14.3% of the Egr3-correlated genes) have a significant *p-value* (≤0.05). A 10,000x simulation was performed using the background percentage of significant negative γ_j_ tumor *p-values* (6.1%) from the SPECS U133Plus2.0 data set. The results of the simulation reveal that the Egr3-correlated gene list contains a significantly higher percentage of significant γ_j_ values than does the background SPECS data set (*p-value = *0.00241). In addition to the 14 probe sets with a significant negative γ_j_ value, another 12 probe sets have a γ_j_ tumor with *p-value* ≤0.1, indicating that Egr3-correlated genes tend to show higher expression in non-relapse than in relapse tumor samples.

In order to test whether these genes contain known Egr3 regulatory sequences (EREs), Genomatix MatInspector was used to analyze the promoter sequences of the Egr3-correlated genes (1500 bases upstream to 1000 bases downstream of the transcription start site). MatInspector promoter analysis revealed that 94% (76 genes) contain one or more putative ERE sites with a matrix similarity of 0.80 or greater. MatInspector grades ERE sequences according to their similarity to the ERE consensus sequence (GCGG/TGGGCG). The matrix similarity score takes into account the conservation of each sequence position of the matrix and gives the most weight to those positions that are conserved. For comparison we examined the frequency of EREs in the Genomatix vertebrate background model. We analyzed the V$EGRF family (the Transfac annotation for the vertebrate EGR family), which includes Egr1, Egr2, Egr3, WT, and CKROX, because the background frequency of binding sites given by MatInspector is for the entire family. MatInspector reports that 62.7% of vertebrate promoters contain a V$EGRF binding site which matches the family matrix with optimized matrix similarity. MatInpector uses the optimized matrix similarity (between 79–92% for V$EGRF family members) as a cutoff for actual binding sites to reduce the number of false positive matches. When we include WT and CKROX in addition to the ERE binding sites already indentified, 99% of the Egr3-correlated genes contain a V$EGRF site. We also performed a simulation where 62.7% of genes from the U133 Plus2.0 Affymetrix expression platform were designated to contain a V$EGRF site and selected 81 genes randomly each time for 10,000 repetitions. We determined that the 10,000 sets of 81 randomly selected genes never contained a V$EGRF site at ≥99% frequency (*p*<0.0001). We conclude that the list of Egr3-correlated genes identified in the prostate cancer gene expression data sets are indeed significantly enriched for potential Egr3 binding sites.

### Egr3-correlated Genes are Enriched in Immunoregulatory Functions Including IL6 and IL8

The Egr3-correlated gene list noticeably clusters into several important functions associated with prostate cancer, such as immune response and proliferation. To further analyze these pathways, we utilized the pathway software MetaCore (Thomson Reuters) and found that many of the genes clustered into common pathways, transcription factor regulatory networks, and functional categories. We were then able to confirm these pathways and networks using the curated literature references. MetaCore recognized 80 of the Egr3-correlated genes as annotated, protein coding genes. Of the 80 genes, 26 were reported to interact with an Egr family member (Egr1, Egr2, or Egr3). Details of these interactions are summarized in **Table 3**.

**Table 3:** Egr3-correlated genes with a reported interaction with an Egr transcription factor*.

Network Object	Affymetrix ID	Effect	Manual Curation	References
Cyr61	201289_at	Activation	ChIP	PMID: 12899698; PMID:16113055; PMID:17975260
IL-6	205207_at	Activation	ChIP	PMID:17498291; PMID:18281687
ATF-3	202672_s_at	Activation	expression, promoter activation, shRNA	PMID:16079301; PMID:16489044; PMID:17975260; PMID:18218726; PMID:18719024; PMID:21205742
EGR3	206115_at	Activation	expression	PMID:14551154; PMID:20506119
IL-1 beta	39402_at	Activation	siRNA, expression	PMID:20363028; PMID:20624458
CD44	217523_at	Activation	reporter plasmid, deletion/mutation construct	PMID:8628295; PMID:9300687; PMID:12670907; PMID:15923644; PMID:19195913
IL-8	202859_x_at	Activation	ChIP, shRNA, Egr1 overexpression	PMID:18281687; PMID:19837667
LDLR	202068_s_at	Activation	ChIP	PMID:12235180; PMID:12947119; PMID:16113055
EGR2 (Krox20)	205249_at	Activation	expression	PMID:19032775; PMID:19374776; PMID:20506119
COX-2 (PTGS2)	204748_at	Activation	EMSA, ChIP, siRNA	PMID:9520467; PMID:16840740; PMID:20546888; PMID:20624458
GADD45 beta	207574_s_at	Activation	ChIP	PMID:19834918
p21	202284_s_at	Activation	EMSA, nuclear pull down, ChIP	PMID:12690110; PMID:15523672; PMID:17307334; PMID:20368687; PMID:20953893
C/EBPdelta	203973_s_at	Activation	ChIP	PMID:19365618
ZFP36 (Tristetraprolin)	201531_at	Activation	reporter and deletion contructs	PMID:7559666; PMID:12556466
NUR77 (NR4A1)	202340_x_at	Activation	EMSA, expression data	PMID:8413214; PMID:9858508
EGR1	201693_s_at	Activation	ChIP, expression	PMID:8065330; PMID:11830539; PMID:19365618; PMID:20018936
CD69	209795_at	Activation	EMSA	PMID:12385031; PMID:14660624
PHLDA1	217996_at	Unspecified		PMID:15315823
PFKFB3	202464_s_at	Unspecified		PMID:10673355
Lamin A/C	212089_at	Unspecified		PMID:18291030
KLF6	208961_s_at	Unspecified		PMID:16054710
EMP1	213895_at	Unspecified		PMID:16113055
HB-EGF	203821_at	Unspecified		PMID:14551154
BTG2	201236_s_at	Unspecified		PMID:18196550
FosB	202768_at	Unspecified		PMID:14551154
Fra-2 (FOSL2)	218880_at	Unspecified		PMID:19032775
GRO-2 (CXCL2)	209774_x_at	Unspecified		PMID:14551154

* Interactions and references are as reported by MetaCore. Genes from this table are highlighted blue **Table S1**.

Because extensive literature exists concerning Egr1-regulated genes and very little exists concerning Egr3-regulated genes, we will use Egr1 as a surrogate in the below analyses. The MetaCore interaction analysis is based on the total reported number of interactions that a given object (protein) has with all other DNA regulatory elements or proteins. Egr1, the best studied Egr family member, has 728 reported interactions with promoters or other transcription factors. Based on this number, it is expected that Egr1 would interact with 2.47 genes from our list of 80 Egr3-correlated genes. Because Egr1 actually interacts with 26 genes, we conclude that our list is significantly enriched with Egr1-interacting genes (*p* = 5.45×10^−20^) and that Egr1 is overconnected (interacts with significantly more genes of the Egr3-correlated gene list than expected given the background model). Since very little has been reported on the function of Egr3 and its interactions, we do not have a long list of Egr3-regulated genes. MetaCore interaction analysis does however identify Egr3 as being overconnected. Egr3 is known to interact with only 52 other genes or proteins, and our list of 80 correlating genes contains 4 interactions (*p* = 2.93×10^−5^). We also note that even though Egr3 has few reported interactions, all Egr family members bind to the same promoter regulatory element (ERE), and we infer that Egr3 could potentially regulate the same promoters as Egr1. The ERE analysis provides supporting evidence that the significant correlation of the steady state mRNA expression of these 80 genes with Egr3, as determined for the fresh frozen prostate cancer cases, represents biologically related genes that are potential target genes of Egr3.

Based on these findings, Metacore was used to examine potential pathways involving the Egr3-correlated genes, including annotated processes, diseases, metabolic networks, and gene ontology processes. The results of the enrichment analysis are summarized in **Table** **4**. The 80 Egr3-correlated genes are enriched in pathways such as gonadatropin releasing hormone signaling, PEDF (serpinF1) signaling, multiple immune response pathways, prostaglandin E2 pathway, and the AP-1 regulation of cellular metabolism pathway. Of particular note are the multiple immune pathways that include genes such as IL-6, IL-8, IL1β, COX2, and IRF1. We also utilized the publicly available gene ontology software David (http://david.abcc.ncifcrf.gov/home.jsp) to further analyze functional connections between our 80 Egr3-correlated genes. Analysis of the 80 unique Egr3-correlated genes in David identified additional functional categories that these genes cluster into. The enriched categories include transcription factor activity (22 genes), blood vessel development (10 genes), response to wounding (15 genes), and regulation of apoptosis (18 genes).

**Table 4:** MetaCore enrichment analysis of Egr3-correlated genes.

GeneGo Pathway Maps	*p* Value	GeneGo Diseases	*p* Value
Reproduction: GnRH signaling	4.3E−09	Inflammation	4.5E−16
Development: PEDF signaling	7.1E−09	Pathologic Processes	2.5E−15
Immune response: IL-1 signaling pathway	1.2E−07	Skin and Connective Tissue Diseases	5.2E−14
Immune response: Histamine signaling in dendritic cells	7.3E−06	Bacterial Infections and Mycoses	1.1E−13
PGE2 pathways in cancer	1.2E−05	Vasculitis	2.6E−13
Immune response: IL-17 signaling pathways	1.8E−05	Infection	3.1E−13
Immune response: CD40 signaling	2.5E−05	Skin Diseases	5.7E−13
Transcription: Role of AP-1 in regulation of cellular metabolism	5.3E−05	Neoplasms by Histologic Type	1.5E−12
Immune response: MIF in innate immunity response	6.5E−05	Rheumatic Diseases	9.2E−12
Immune response: PGE2 signaling in immune response	1.0E−04	Virus Diseases	1.0E−11
**GeneGo Process Networks**	***p*** ** Value**	**Gene Ontology Processes**	***p*** ** Value**
Reproduction: Gonadotropin regulation	2.4E−08	response to organic substance	6.6E−23
Cell adhesion: Platelet-endothelium-leucocyte interactions	4.0E−06	positive regulation of biological process	3.3E−19
Chemotaxis	4.1E−06	regulation of biological process	1.8E−17
Proliferation: Negative regulation of cell proliferation	6.0E−06	regulation of cellular process	2.7E−17
Immune response: Th17-derived cytokines	3.0E−05	negative regulation of cellular process	3.5E−17
Inflammation: Interferon signaling	6.4E−05	response to stress	4.0E−17
Inflammation: Histamine signaling	7.7E−04	developmental process	4.9E−17
Reproduction: GnRH signaling pathway	8.1E−04	biological regulation	5.3E−17
Inflammation: IL-10 anti-inflammatory response	1.2E−03	response to chemical stimulus	6.1E−17
Apoptosis: Anti-Apoptosis mediated by external signals via PI3K/AKT	1.3E−03	positive regulation of cellular process	8.0E−17
**GeneGo Metabolic Networks**	***p*** ** Value**		
Pentose phosphate pathways and transport	3.8E−06		
GalNAcbeta1-3Gal pathway	5.4E−03		
phosphatidylethanolamine pathway	5.0E−02		
Carbohydrate metabolism: Fructose metabolism and transport	6.5E−02		
Vitamin, mediator and cofactor metabolism: Alpha-tocotrienol	2.0E−01		
Decanoylcarnitine pathway	2.9E−01		
O-hexanoyl-(L)-carnitine pathway	3.0E−01		
Acyl-L-carnitine pathway	3.0E−01		
Lauroylcarnitine pathway	3.0E−01		
Glycine pathway	3.0E−01		

### External Validation of Expression

As an external validation of our list, we analyzed GEO dataset GDS2545, which contains gene expression data for 171 prostate samples. The samples include normal prostate tissue from donors, tumor-adjacent tissue, primary prostate cancer tissue, and metastatic tissue. The experiments published by Chandran et al. [27] and Yu et al. [26] were performed on the Affymetrix U95Av2 platform, which includes gene expression data for 12,625 probe sets. We downloaded the publicly available data and carried out the same correlating gene analysis on this data as we did on our own Affymetrix data. Importantly, the Egr3 mRNA expression profile was very similar to what is seen in the SPECS prostate gene expression data. Egr3 (probe set 40375_at) exhibits the same pattern as was seen in the SPECS data set where expression was low in normal donor prostate tissue, increased in tumor-adjacent normal tissue, increased in primary prostate tissue, and decreased in metastatic samples. The GEO Profiles histogram of Egr3 expression for this dataset is shown in **Figure S3**.

From the downloaded dataset, the top 150 Egr3-correlated probe sets (121 unique genes) based on the Pearson correlation *p*-value were compared to our list of 98 correlating probe sets (80 unique genes). The top 150 Egr3-correlated probe sets had a Pearson’s correlation coefficient cutoff of 0.43 and a Pearson’s *p-value* cutoff of 5.18×10^−9^. The overlap between the two lists is 43 unique genes. Using the same simulation approach as was used for other comparisons, we randomly selected groups of 80 and 121 genes from a total of 9148 genes (the number of unique genes on the U95Av2 platform). After 10,000 simulations it was determined that the 80 and 121 genes never overlap by 43 or more based on random chance alone (*p*<.0001). By performing this analysis on an independent dataset and a different Affymetrix platform, we have validated that our list of 80 genes is reproducibly associated with Egr3 expression in prostate cancer.

### Cell Biology Validation of Il6 and Il8 Regulation of Expression by Egr3

**Figure 3:**
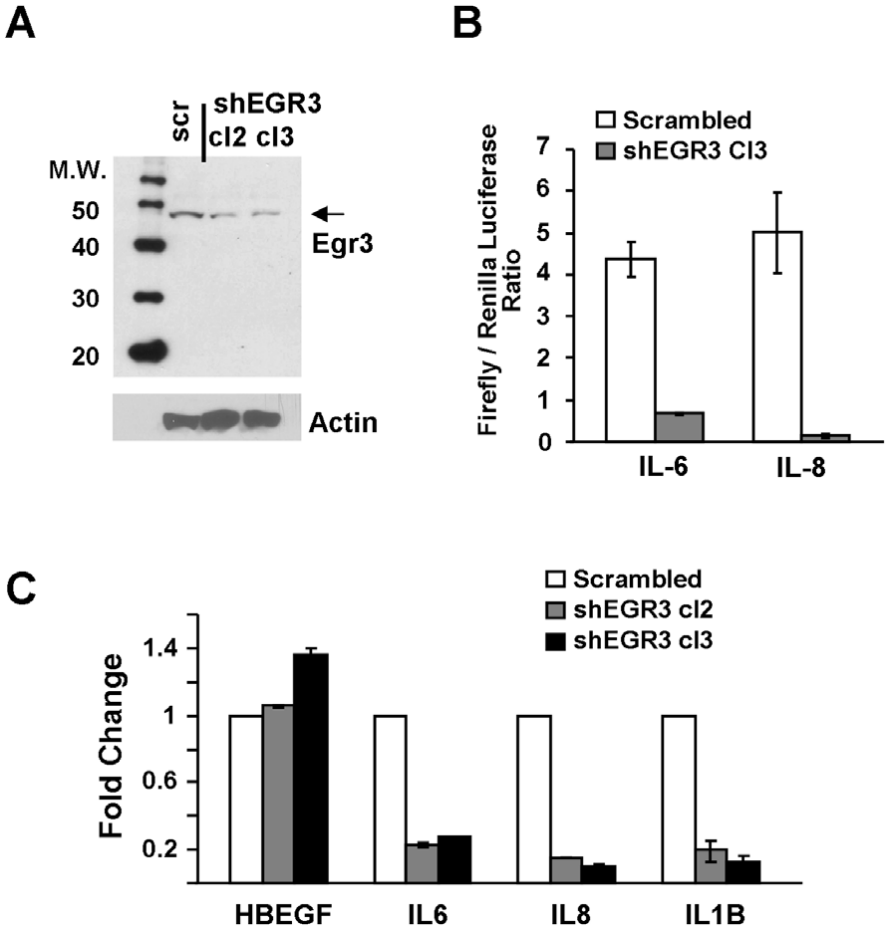
Egr3 target validation. **A:** Stable Egr3 knockdown in M12 prostate cancer cells. M12 cells were transfected with scramble or shEgr3 plasmids and subjected to antibiotic selection to achieve stable knockdown. The shSCR-M12 and two shEgr3-M12 clones (cl2 and cl3) were used for further experiments. Egr3 protein levels were analyzed by western blotting using anti-Egr3 antibodies. Membranes were stripped and reprobed with antibodies to β-actin. Molecular weights are shown on the left. **B:** shSCR-M12 and shEgr3-M12 clone 3 were transfected with pGL3-IL6 or pGL3-IL8 reporter plasmids and a renilla luciferase plasmid as described in Methods. A commercial luciferase assay was used to measure firefly and renilla luciferase activity. Results show the ratio of IL6 and IL8 promoter-dependent firefly luciferase activity normalized to renilla for each condition. **C:** qPCR analysis of Egr3 target genes. Total RNA was extracted from M12 (scramble) and M12 shEgr3 (cl2 and cl3) and analyzed by quantitative RT-PCR. Expression levels were assessed using the 2–ΔΔCT relative quantification method and GAPDH was used for normalization. Results as expressed as function of control (scramble). The error bars were generated using the 2–ΔΔCT method to take into account the standard deviation of GAPDH and the standard deviation of the measured gene.

To provide biological validation of our findings, we achieved stable knockdown of Egr3 expression in M12 prostate cancer cells. These cells were chosen because of their high expression of Egr3 (M12 cells are derived from the P69 cell line, which is not tumorigenic and displays undetectable Egr3 expression - data not shown). As shown in **Figure 3A**, Egr3 protein expression was decreased by half in shEgr3-M12 cells compared to control (scramble) M12 cells, as shown by western blot analysis. We then focused on inflammation mediators IL6 and IL8 because of their known role in prostate cancer and their distinct expression pattern in relapse and non-relapse tumors that correlates with that of Egr3. In one set of experiments, a reporter assay was performed in shSCR-M12 or shEgr3-M12 cells, using promoter sequences for IL6 and IL8. **Figure 3B** shows that the reporter luciferase activity dependent on each promoter was inhibited in shEgr3-M12 cells, indicating that Egr3 indeed regulates the promoter of these genes in M12 cells (t-test, *p* = 0.0019 for IL-6 and 0.0063 for IL-8). In another set of experiments, the mRNA level of IL6, IL8 and two other genes (HBEGF and IL1B) was measured by real-time qRT-PCR. Gene expression was compared between shSCR-M12 and shEgr3-M12 cells, as shown in **Figure 3C**. Although expression of HBEGF was not altered in shEgr3-M12 cells, the expression of IL6, IL8 and IL1B was strongly inhibited, again indicating that Egr3 controls the expression of these cytokines in prostate cancer cells M12. It is interesting to note that HBEGF is a target of Egr1 in prostate cancer cells [41], and it is therefore possible that the correlation of HBEGF with Egr3 was due to its regulation by Egr1, which itself was highly correlated with Egr3 (**Table S1**).

We conclude that IL6 and IL8, identified as Egr3 potential target genes using in silico analysis, are indeed regulated by Egr3 in prostate cancer cells.

## Discussion

Thigpen el al. [14] and Eid et al. [15] reported in the 1990s that Egr1 mRNA and protein are over-expressed in prostate cancer. Until now, Egr3 expression has not been examined in prostate cancer even though these two transcription factors are regulated by many of the same stimuli and bind the same DNA response element. We are the first to describe the over-expression of Egr3 mRNA and protein in prostate cancer. Egr3 RNA and protein are expressed predominantly in the epithelial cells in both normal prostate glands and prostate cancer. Through multiple linear regression analysis of Egr3 expression in specific cell types (tumor, stroma, BPH) we were also able to show that Egr3 mRNA is up-regulated in prostate cancer of men with less aggressive disease (non-relapse) but *not* in men who will eventually go on to relapse. We would like to emphasize two aspects of the SPECS study that we believe are of particular importance. One is that the tumor samples were obtained early on, at the time of prostatectomy, while relapse status was observed later on during follow up. Thus, low expression of Egr3 (and Egr3-correlated genes) in a sub-group of tumors is an early marker of tumors that *will* relapse as opposed to tumors with high Egr3 expression, which do not relapse. It is not, however a measure of progression as defined by Gleason scores or grades, since there was no significant difference in Gleason scores between the relapse and non-relapse groups at the time of surgery (median = 7 for both groups; average Gleason = 7 in relapse, average Gleason = 6.7 in non-relapse, the difference was not statistically significant). This also explains that there was no correlation between Egr3 and Gleason score.

Another aspect is that changes in patterns of expression, as evidenced in this study, may be masked when the composition of samples is not analyzed. Egr3, for example, is strongly expressed in epithelial tumor cells but not in other stromal cells, therefore observed changes in expression in tumor tissue reflect mostly changes in tumor cells. However, when a gene is expressed strongly in the stroma and this expression does not vary between normal and cancer, changes in expression that may occur in epithelial tumor cells may not be effectively detected because they would be masked by the strong stromal expression. Our analysis allows identification of cell type-specific patterns, which is its uniqueness and its main strength.

Our results suggest that Egr3 may act both as a diagnostic marker (increased expression in cancer compared to normal) and as a prognostic marker that may distinguish between aggressive and non-aggressive tumors (differentially expressed in non-relapse and relapse). This distinct expression pattern of Egr3 is intriguing and warrants further study. Indeed, understanding the molecular mechanism behind the regulation of its expression could shed some light on its relevance in prostate cancer.

The nature of Egr3 actions in prostate cancer is not yet clear; however some insight into its function can be gained through analysis of the genes whose expression correlates with it. Since Egr3 is a transcription factor, it is plausible that it directly regulates the expression of a number of these genes, and it may also indirectly regulate the expression of others. The list of Egr3-correlated genes is significantly enriched with genes that are more highly expressed in non-relapse prostate tumor cells than in relapse prostate tumor cells, following the same expression profile as Egr3, and leading us to conclude that Egr3 transcription factor activity may be responsible for the differential regulation of these genes in non-aggressive and aggressive disease.

Potential Egr3 target genes include an extensive list of cytokines, chemokines, and other inflammation-associated genes such as IL6, IL8, IL1β, CCL3, CCL4 (MIP1b), CXCL2, SOCS3, COX2, CD69, and CD44. We reported the presence of Egr binding sites in most of the Egr3correlated genes, and indeed many of these genes have been reported as direct Egr targets in the literature. Of the inflammatory genes, IL8, IL1β, IL6, CXCL2, CD69, CD44, and COX2 are *known* to be Egr1 target genes through the use of chromatin immunoprecipitation, reporter assays, siRNA/shRNA, and electromobility shift assays [42]–[49]. For example IL6 and IL8, which we identify and validate as Egr3 targets, are also regulated by Egr1. Treatment of human carcinoma KB cells with Egr1 siRNA led to a decrease in IL6 and IL8 promoter activation, and Egr1 was found to bind to the promoters of both interleukins [43]. Egr1 also regulates IL8 in the DU145 prostate cancer cell line, as Egr1 shRNA caused a decrease in IL8 transcription and production [42].

As noted previously, Egr1 and Egr3 bind to similar sequence motifs on promoters and it is therefore unsurprising that they share a set of target genes such as IL6 and IL8, which we have now validated as direct targets for Egr3 in prostate cancer cells. The distinction between relapse and non-relapse observed for Egr3 and Egr3-correlated genes (among which Egr1, Il6 and IL8) has not been made previously. We suggest that their low expression at the time of surgery (as opposed to high expression in most cancer samples) distinguishes more aggressive tumors, where aggressiveness is defined by relapse, not by Gleason score or grade.

IL6 and IL8 are of particular interest because of the rich literature concerning these interleukins and prostate cancer. IL6 was increased in the serum of men with advanced prostate cancer [50]. It was later found to act as an autocrine growth factor in prostate cancer cells, and IL6 protein concentration was found to be up-regulated 18 fold in prostate cancer tissue compared to normal prostate tissue [51]. Recently, a role for IL6 in prostate tumorigenesis and progression was reported. Treatment of benign cells with the cytokine led to increased proliferation and an epithelial-mesenchymal transition (EMT) phenotype, which suggests a role for IL6 in the earlier stages of prostate cancer [52]. IL8 is a pro-angiogenic chemokine (CXCL8) that is over-expressed in prostate cancer epithelial cells compared to normal prostate glands [53]. Treatment of DU145 and PC3 prostate cancer cell lines with IL8 siRNA led to cell cycle arrest, increased apoptosis, and enhanced chemotherapeutic efficacy [54].

NFκB is a well known regulator of the inflammatory response, and it is known to act as cofactor for Egr transcription factors in the regulation of many inflammatory genes. Egr3 physically interacts with NFκB subunits p50 and p65, and Egr3-p65 complexes strongly activate the IL2, TNFα, and ICAM1 promoters [55]. Although the amount of literature detailing Egr3 regulation of inflammatory genes is small, previous observations show that the NFκB-Egr3 complex has the potential to regulate many inflammatory genes important to cancer.

The discovery of Egr3 regulation of these interleukins could potentially broaden our understanding of inflammation and the immune system in prostate cancer.

While the in-silico approach we used to examine Egr3 expression and possible target genes is very informative, much work remains to be done in order to determine Egr3 mode of action and target genes in prostate cancer. Egr3 mRNA exhibited an intriguing expression pattern in normal, non-relapse and relapse samples, which requires further investigation. Egr3-null mice do exist, and the result of crossing these mice with a prostate cancer model such as TRAMP or PTEN-deficient mice would be of great interest. Analysis of Egr3 function in a prostate cell model system is in progress, and the results of this analysis will help shed light on Egr3’s gene regulatory activities in prostate cancer and possible reasons for Egr3 expression pattern in prostate cancer.

## Supporting Information

Figure S1 **HPA Egr3-Labeled** **Prostate Tumor Samples.** Human Protein Atlas anti-Egr3 immunohistochemistry (left) and Aperio ImageScope pseudocolored prostate sections (right) for all available prostate tumor samples based on thresholding as described in Materials and Methods.
(PDF)

Figure S2 **HPA Egr3-Labeled Normal Prostate Samples.** Human Protein Atlas anti-Egr3 immunohistochemistry (left) and Aperio ImageScope pseudocolored prostate sections (right) for all available normal prostate sections based on thresholding as described in Materials and Methods.
(PDF)

Figure S3 **Geo Profiles histogram of Egr3 expression for GEO dataset GDS2545.** Egr3 expression for normal prostate, tumor adjacent normal prostate, primary prostate cancer, and metastatic prostate cancer (from left to right). The left axis (red bars) represents the expression value and the right axis (blue squares) represents the % rank for the expression of the probe set compared to all other genes on the array. A larger histogram can be vied at the NCBI GEO website http://www.ncbi.nlm.nih.gov/sites/GDSbrowser?acc=GDS2545 by searching for Egr3 under “data analysis tools.”
(PDF)

Figure S4 **Primers.** Sequence of the primers used in the real-time qPCR experiments.
(PDF)

Table S1 Genes whose expression correlates with the distinct expression pattern of Egr3 in relapse and non-relapse prostate cancer (n = 263; R p-value≤0.001)*.
(PDF)
