# Supplementary material for: Early Growth Response 3 (Egr3) Is Highly Over-Expressed in Non-Relapsing Prostate Cancer but Not in Relapsing Prostate Cancer
Source: PLoS One. 2013 Jan 14;8(1):e54096. doi: 10.1371/journal.pone.0054096 (PMC3544741; doi:10.1371/journal.pone.0054096)
Supplement: Figure S1 — HPA Egr3-Labeled Prostate Tumor Samples. Human Protein Atlas anti-Egr3 immunohistochemistry (left) and Aperio ImageScope pseudocolored prostate sections (right) for all available prostate tumor samples based on thresholding as described in Materials and Methods. (PDF) [file pone.0054096.s001.pdf]

Figure S1

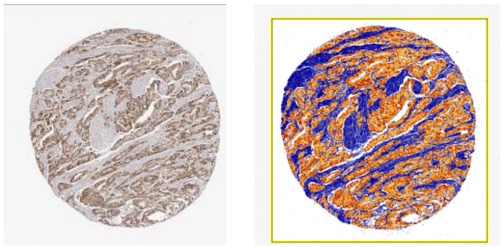

Patient 3303(1)

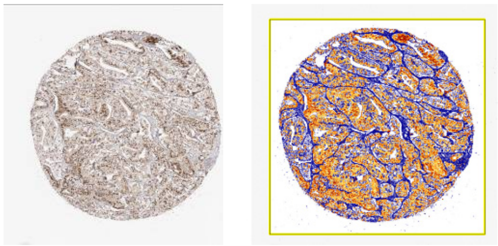

Patient 3359(1)

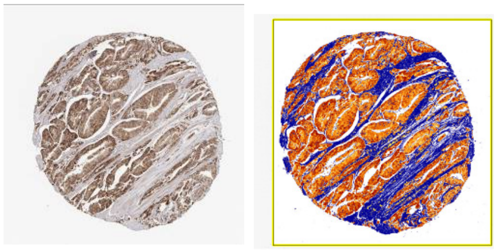

Patient 3303(2)

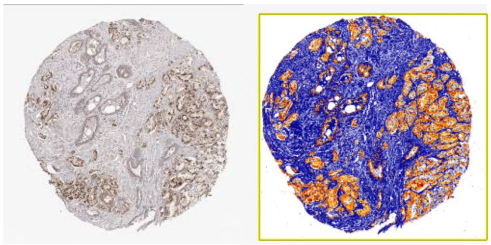

Patient 3359(1)

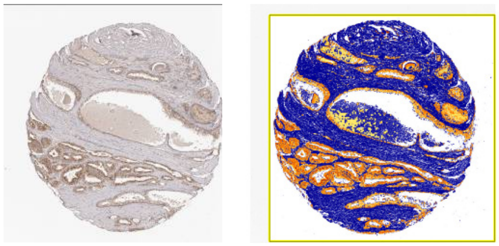

Patient 3558(1)

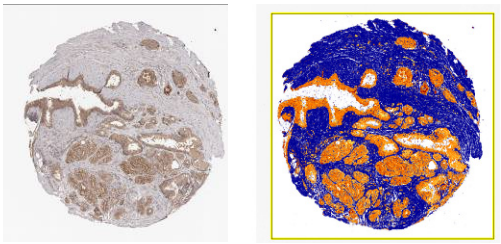

Patient 3570(1)

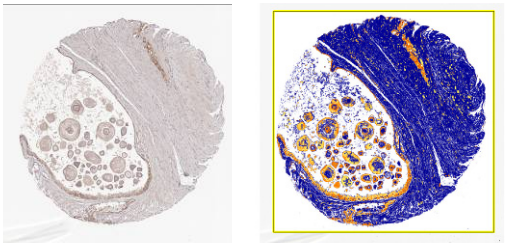

Patient 3558(2)

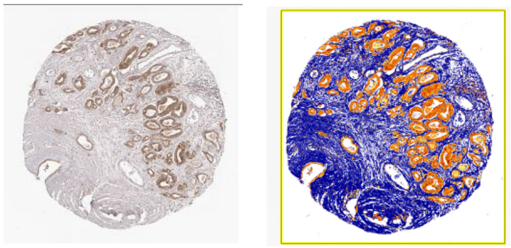

Patient 3748(1)

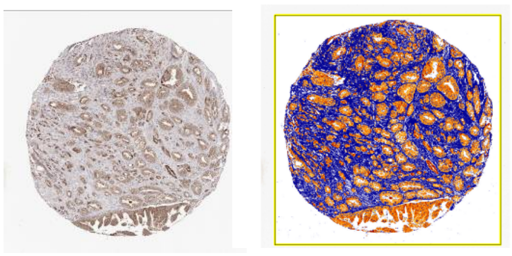

Patient 3579(1)

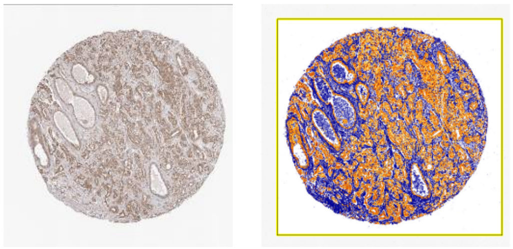

Patient 3571(1)

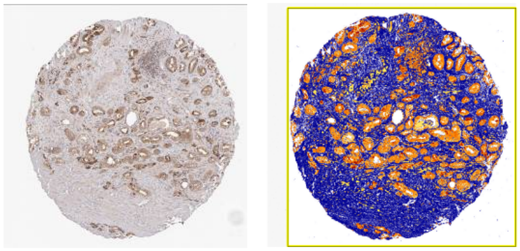

Patient 3579(2)

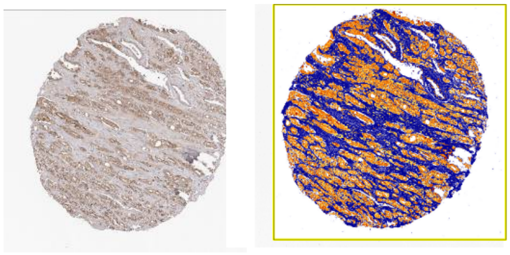

Patient 3571(2)

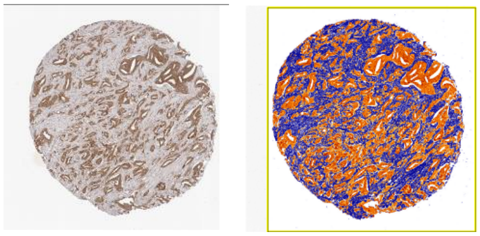

Patient 3744(1)

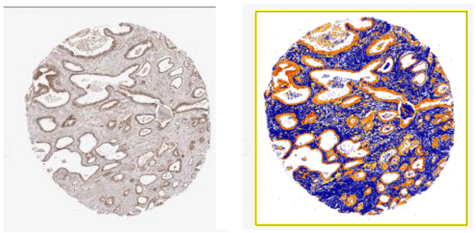

Patient 3742(1)

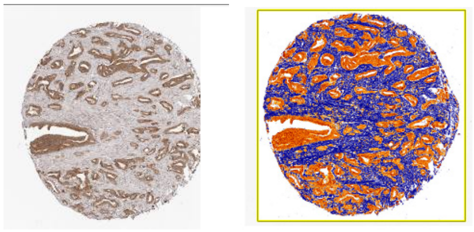

Patient 3744(2)

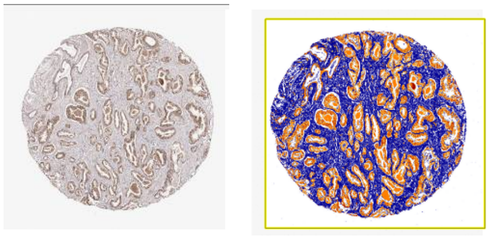

Patient 3742(2)

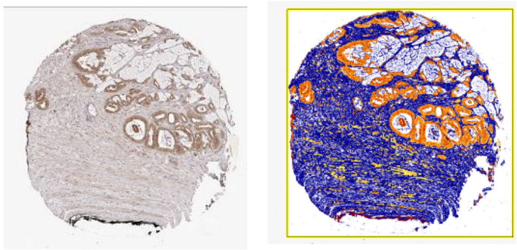

Patient 3747(1)

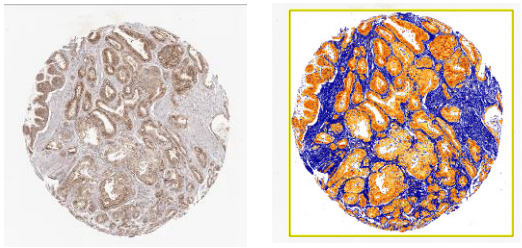

Patient 3746(1)

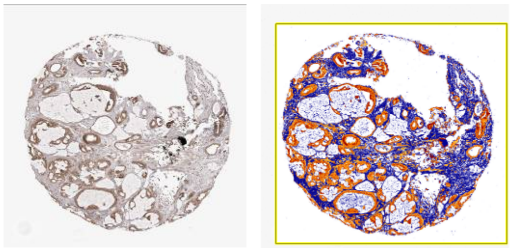

Patient 3747(2)

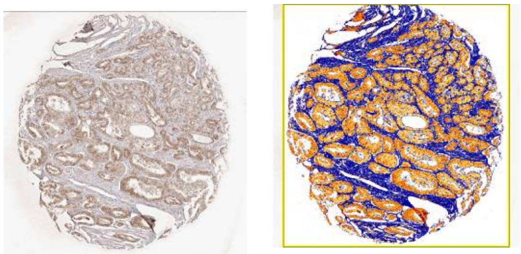

Patient 3746(2)
