# Supplementary figures and images for: Early Growth Response 3 (Egr3) Is Highly Over-Expressed in Non-Relapsing Prostate Cancer but Not in Relapsing Prostate Cancer
Source: PLoS One. 2013 Jan 14;8(1):e54096. doi: 10.1371/journal.pone.0054096 (PMC3544741; doi:10.1371/journal.pone.0054096)

**Figure S2**

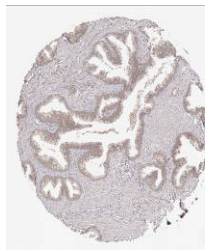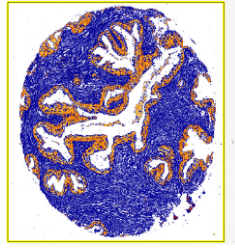

Patient 2098 Normal

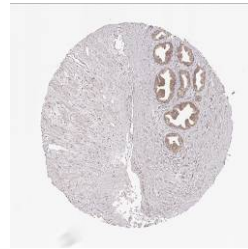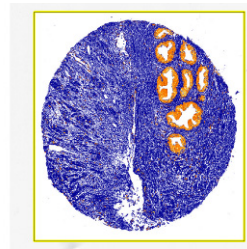

Patient 2472 Normal

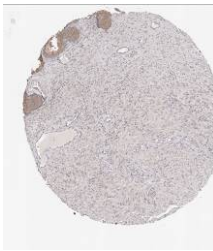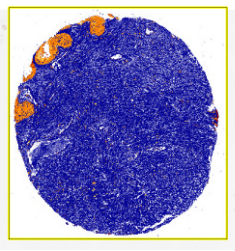

Patient 2932

Supplement: Figure S2 — HPA Egr3-Labeled Normal Prostate Samples. Human Protein Atlas anti-Egr3 immunohistochemistry (left) and Aperio ImageScope pseudocolored prostate sections (right) for all available normal prostate sections based on thresholding as described in Materials and Methods. (PDF) [file pone.0054096.s002.pdf]

Figure S3

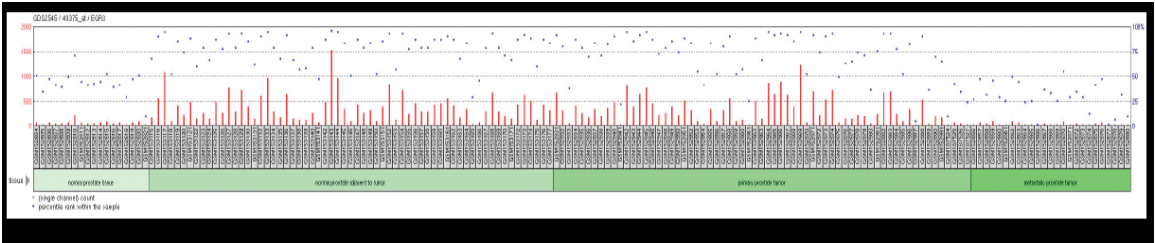

Supplement: Figure S3 — Geo Profiles histogram of Egr3 expression for GEO dataset GDS2545. Egr3 expression for normal prostate, tumor adjacent normal prostate, primary prostate cancer, and metastatic prostate cancer (from left to right). The left axis (red bars) represents the expression value and the right axis (blue squares) represents the % rank for the expression of the probe set compared to all other genes on the array. A larger histogram can be vied at the NCBI GEO website http://www.ncbi.nlm.nih.gov/sites/GDSbrowser?acc=GDS2545 by searching for Egr3 under “data analysis tools.” (PDF) [file pone.0054096.s003.pdf]
