# Supplementary material for: Early Growth Response 3 (Egr3) Is Highly Over-Expressed in Non-Relapsing Prostate Cancer but Not in Relapsing Prostate Cancer
Source: PLoS One. 2013 Jan 14;8(1):e54096. doi: 10.1371/journal.pone.0054096 (PMC3544741; doi:10.1371/journal.pone.0054096)
Supplement: Table S1 — Genes whose expression correlates with the distinct expression pattern of Egr3 in relapse and non-relapse prostate cancer (n = 263; R p-value≤0.001)*. (PDF) [file pone.0054096.s005.pdf]

Table S1: Genes whose expression correlates with the distinct expression pattern of Egr3 in relapse and non-relapse prostate cancer (n=263; R p-value≤ 0.001) *

Gene Name	Probe ID	Description	R value U133A Dataset	R value U133 Plus2 Dataset	Slope U133 Plus2 Dataset
FOSB	202768_at	FBJ murine osteosarcoma viral oncogene	0.806	0.917	1.354
SELE	206211_at	selectin E (endothelial adhesion molecule 1)	0.805	0.864	1.059
IL6	205207_at	interleukin 6 (interferon, beta 2)	0.603	0.774	1.052
ATF3	202672_s_at	activating transcription factor 3	0.759	0.906	1.011
EGR3	206115_at	early growth response 3	1.000	1.000	1.000
EGR1	201693_s_at	early growth response 1	0.628	0.893	0.939
NR4A3	207978_s_at	nuclear receptor subfamily 4, group A, member 3	0.605	0.825	0.925
EGR2	205249_at	early growth response 2 (Krox-20 homolog, Drosophila)	0.859	0.932	0.914
NR4A3	209959_at	nuclear receptor subfamily 4, group A, member 3	0.721	0.842	0.909
AREG	205239_at	amphiregulin (schwannoma-derived growth factor)	0.539	0.760	0.894
NR4A2	216248_s_at	nuclear receptor subfamily 4, group A, member 2	0.721	0.892	0.848
NR4A2	204621_s_at	nuclear receptor subfamily 4, group A, member 2	0.601	0.871	0.831
FOSL1	204420_at	FOS-like antigen 1	0.563	0.702	0.810
NR4A2	204622_x_at	nuclear receptor subfamily 4, group A, member 2	0.747	0.889	0.770
CXCL2	209774_x_at	chemokine (C-X-C motif) ligand 2	0.607	0.661	0.758
SOD2	215078_at	superoxide dismutase 2, mitochondrial	0.575	0.516	0.744
IL8	202859_x_at	interleukin 8	0.633	0.536	0.738
SOCS3	206359_at	suppressor of cytokine signaling 3	0.622	0.822	0.690
CEBPD	213006_at	CCAAT/enhancer binding protein (C/EBP), delta	0.695	0.776	0.676
CCL4	204103_at	chemokine (C-C motif) ligand 4	0.621	0.683	0.671
IL1B	39402_at	interleukin 1, beta	0.542	0.730	0.663
CCL3 // CCL3L1 // CCL3L3	205114_s_at	chemokine (C-C motif) ligand 3 / chemokine (C-C motif) ligand 3-like 1 /chemokine (C-C motif) ligand 3-like 3	0.629	0.569	0.657
ZFP36	201531_at	zinc finger protein 36, C3H type, homolog (mouse)	0.675	0.884	0.636
HBEGF	203821_at	heparin-binding EGF-like growth factor	0.759	0.849	0.621
LMLN	38037_at	Leishmanolysin-like (metallopeptidase M8 family)	0.763	0.841	0.614
GADD45B	209305_s_at	growth arrest and DNA-damage-inducible, beta	0.599	0.718	0.611
CD69	209795_at	CD69 antigen (p60, early T-cell activation antigen)	0.571	0.641	0.603
GADD45B	209304_x_at	growth arrest and DNA-damage-inducible, beta	0.679	0.827	0.602

PHLDA1	217996_at	pleckstrin homology-like domain, family A, member 1	0.598	0.798	0.587
NR4A1	202340_x_at	nuclear receptor subfamily 4, group A, member 1	0.759	0.800	0.581
GADD45B	207574_s_at	growth arrest and DNA-damage-inducible, beta	0.736	0.835	0.580
SLC2A3	202497_x_at	solute carrier family 2 (facilitated glucose transporter), member 3	0.536	0.806	0.572
SLC2A3	202499_s_at	solute carrier family 2 (facilitated glucose transporter), member 3	0.692	0.885	0.555
JUNB	201473_at	jun B proto-oncogene	0.697	0.730	0.550
CYR61	201289_at	cysteine-rich, angiogenic inducer, 61	0.581	0.818	0.547
DKFZP434F0318	221031_s_at	hypothetical protein DKFZp434F0318 /// hypothetical protein DKFZp434F0318	0.686	0.797	0.533
EMP1	213895_at	epithelial membrane protein 1	0.600	0.746	0.525
MAFF	205193_at	v-maf musculoaponeurotic fibrosarcoma oncogene homolog F (avian)	0.778	0.765	0.499
EIF1	212225_at	eukaryotic translation initiation factor 1	0.563	0.689	0.492
KLF4	220266_s_at	Kruppel-like factor 4 (gut)	0.601	0.652	0.491
ZIC4	36711_at	Zic family member 4	0.834	0.774	0.479
PTGS2	204748_at	prostaglandin-endoperoxide synthase 2 (prostaglandin G/H synthase and cyclooxygenase)	0.555	0.738	0.479
IER3	201631_s_at	immediate early response 3	0.550	0.762	0.474
KLF6	208961_s_at	Kruppel-like factor 6	0.669	0.607	0.466
KLF6	208960_s_at	Kruppel-like factor 6	0.549	0.594	0.466
PPP1R15A	202014_at	protein phosphatase 1, regulatory (inhibitor) subunit 15A	0.668	0.758	0.465
MCL1	200798_x_at	myeloid cell leukemia sequence 1 (BCL2-related)	0.612	0.810	0.459
KLF4	221841_s_at	Kruppel-like factor 4 (gut)	0.801	0.711	0.458
TRIB1	202241_at	tribbles homolog 1 (Drosophila)	0.584	0.701	0.458
THBD	203887_s_at	thrombomodulin	0.609	0.825	0.455
MCL1	214056_at	Myeloid cell leukemia sequence 1 (BCL2-related)	0.658	0.821	0.450
EGR4	207768_at	early growth response 4	0.549	0.558	0.443
---	37028_at	---	0.648	0.765	0.438
EGR1	201694_s_at	early growth response 1	0.654	0.887	0.435
BTG2	201236_s_at	BTG family, member 2	0.629	0.845	0.426
MGC14376	214696_at	hypothetical protein MGC14376	0.626	0.763	0.414
CDKN1A	202284_s_at	cyclin-dependent kinase inhibitor 1A (p21, Cip1)	0.666	0.769	0.413
NR4A1	211143_x_at	nuclear receptor subfamily 4, group A, member 1	0.761	0.821	0.383
TIPARP	212665_at	TCDD-inducible poly(ADP-ribose) polymerase	0.636	0.734	0.379
KLF10	202393_s_at	Kruppel-like factor 10	0.661	0.778	0.359
EMP1	201325_s_at	epithelial membrane protein 1	0.715	0.684	0.358
TNFAIP3	202644_s_at	tumor necrosis factor, alpha-induced protein 3	0.608	0.746	0.350
CD200	209583_s_at	CD200 antigen	0.552	0.678	0.347

CEBPD	203973_s_at	CCAAT/enhancer binding protein (C/EBP), delta	0.618	0.735	0.347
SOX17	219993_at	SRY (sex determining region Y)-box 17	0.708	0.710	0.345
IRF1	202531_at	interferon regulatory factor 1	0.640	0.702	0.344
TNFAIP3	202643_s_at	tumor necrosis factor, alpha-induced protein 3	0.593	0.738	0.329
LMNA	212089_at	lamin A/C	0.568	0.676	0.323
FOSL2	218880_at	FOS-like antigen 2	0.549	0.687	0.319
IER2	202081_at	immediate early response 2	0.650	0.624	0.318
NFIL3	203574_at	nuclear factor, interleukin 3 regulated	0.613	0.672	0.309
PER1	202861_at	period homolog 1 (Drosophila)	0.571	0.577	0.306
PTGER4	204897_at	prostaglandin E receptor 4 (subtype EP4)	0.586	0.482	0.305
ADM	202912_at	adrenomedullin	0.575	0.729	0.302
LDLR	202068_s_at	low density lipoprotein receptor (familial hypercholesterolemia)	0.579	0.608	0.281
DSCR1	208370_s_at	Down syndrome critical region gene 1	0.556	0.722	0.258
WEE1	212533_at	WEE1 homolog (S. pombe)	0.552	0.660	0.258
EMP1	201324_at	epithelial membrane protein 1	0.754	0.684	0.255
RND3	212724_at	Rho family GTPase 3	0.550	0.649	0.250
ITPKC	213076_at	inositol 1,4,5-trisphosphate 3-kinase C	0.570	0.568	0.212
PER2	205251_at	period homolog 2 (Drosophila)	0.665	0.610	0.212
SPRY1	212558_at	sprouty homolog 1, antagonist of FGF signaling (Drosophila)	0.590	0.758	0.212
CCNL1	220046_s_at	cyclin L1	0.687	0.486	0.210
SERPINB9	209723_at	serpin peptidase inhibitor, clade B (ovalbumin), member 9	0.570	0.623	0.210
MCL1	200797_s_at	myeloid cell leukemia sequence 1 (BCL2-related)	0.690	0.690	0.199
DUSP14	203367_at	dual specificity phosphatase 14	0.534	0.470	0.196
SELP	206049_at	selectin P (granule membrane protein 140kDa, antigen CD62)	0.649	0.679	0.174
KIAA0063	201751_at	KIAA0063 gene product	0.600	0.589	0.173
PRG1	201859_at	proteoglycan 1, secretory granule	0.579	0.618	0.173
CD44	217523_at	CD44 antigen (homing function and Indian blood group system)	0.605	0.482	0.168
BCL6	203140_at	B-cell CLL/lymphoma 6 (zinc finger protein 51) /// B-cell CLL/lymphoma 6 (zinc finger protein 51)	0.678	0.638	0.155
FY	208335_s_at	Duffy blood group, chemokine receptor	0.539	0.553	0.139
PBEF1	217739_s_at	pre-B-cell colony enhancing factor 1	0.590	0.475	0.136
AHR	202820_at	aryl hydrocarbon receptor	0.563	0.525	0.134
PFKFB3	202464_s_at	6-phosphofructo-2-kinase/fructose-2,6-biphosphatase 3	0.552	0.697	0.127
PELI1	218319_at	pellino homolog 1 (Drosophila)	0.556	0.446	0.124
ZC3H12A	218810_at	zinc finger CCCH-type containing 12A	0.589	0.496	0.076
DDX3Y	205000_at	DEAD (Asp-Glu-Ala-Asp) box polypeptide 3, Y linked	0.541	0.455	0.069

*Blue highlighted genes have a reported Egr interaction as referenced in Table 4.
